# A mixed-method study on the provision of remote consultations for non-communicable disease patients during the first wave of the COVID-19 pandemic in Latvia: lessons for the future

**DOI:** 10.1186/s12913-022-07634-x

**Published:** 2022-02-26

**Authors:** Mirdza Kursīte, Inese Stars, Ieva Strēle, Inese Gobiņa, Anda Ķīvīte-Urtāne, Daiga Behmane, Alina Dūdele, Anita Villeruša

**Affiliations:** 1 Institute of Public Health, Riga Stradiņš University, Riga, Latvia; 2 Department of Public Health and Epidemiology, Riga Stradiņš University, Riga, Latvia

**Keywords:** Remote consultations, Non-communicable diseases, COVID-19, Mixed-method

## Abstract

**Background:**

The COVID-19 pandemic has challenged the ability of healthcare systems to ensure the continuity of health services for patients with non-communicable diseases (NCDs). The issue of remote consultations has emerged. Before the COVID-19 pandemic, remote consultations were not routinely provided or covered by public health funding in Latvia. This study aimed to describe the dynamics of consultations and the volume of remote consultations provided for patients with particular NCD and explore clinicians’ experiences of providing remote consultations during the first wave of the COVID-19 pandemic in Latvia.

**Methods:**

A mixed-method study focusing on the first wave of the COVID-19 pandemic in Latvia in Spring 2020 was conducted. Quantitative data from the National Health Services were analysed to assess the dynamics of consultations for patients with selected NCDs. Qualitative data were collected through 34 semi-structured interviews with general practitioners (GPs) and specialists and were analysed using an inductive thematic analysis. Purposive maximum variation sampling was used for participant selection.

**Results:**

During the period with the strongest restrictions of scheduled on-site consultations, a decrease in the total number of consultations was observed for a variety of NCDs. A significant proportion of consultations in this period were provided remotely. GPs provided approximately one-third of cancer-related consultations and almost half of consultations for the other selected conditions remotely. Among specialists, endocrinologists had the highest proportion of remote consultations (up to 72.0%), while urologists had the lowest (16.4%). Thematic analysis of the semi-structured interviews revealed five themes: 1) Adjusting in a time of confusion and fear, 2) Remote consultations: safety versus availability, 3) Sacrifice and loss of privacy, 4) Advantages and disadvantages of communication technologies, and 5) Different form of communication and a health literacy challenge.

**Conclusions:**

During the first wave of the COVID-19 pandemic in Latvia, disruptions to health care services decreased the total number of consultations for patients with NCDs provided by both GPs and specialists. In this period, remote consultations proved to be an important instrument for ensuring the continuity of health care for patients with NCDs, and the necessity to develop a well-designed system for telemedicine in Latvia was highlighted.

## Background

Existing evidence suggests the high burden of the COVID-19 pandemic on patients with non-communicable diseases (NCDs). Individuals with NCDs have a higher risk of COVID-19, but pandemic control policies have resulted in significant disruptions to the screening, treatment, and surveillance of NCD patients [1–3]. In May 2020, 155 of the WHO Member States participated in a rapid assessment of the impact of the COVID-19 pandemic on NCDs resources and services. Approximately ¾ of countries reported a considerable degree of disruption to NCD services [4]. The need to maintain continuity of care for patients with NCDs to ensure the optimum level of disease control and management has been recognized as a priority during the COVID-19 pandemic, requiring adjustments to daily routines and the development of new care models.

In Latvia, 14-day COVID-19 cases and death notification rates were among the lowest in the EU countries during the first half of 2020 [5]. However, upon assessment of the epidemiological risks in neighbouring and other EU countries, a state of emergency due to the first wave of the COVID-19 pandemic throughout the country was declared from 12 March until 9 June 2020 [6]. Along with other regulations, on 27 March, it was decided to restrict planned inpatient health care services and secondary outpatient health care services, with only lifesaving health care services and those that required continuity of treatment being offered. Disruptions of planned health care services raised concerns about chronic patients’ access to care. Premature mortality from chronic conditions such as malignant neoplasms, diabetes mellitus, ischaemic heart disease, and chronic lower respiratory disease is one of the highest in Latvia among the EU member states [7].

The potential for remote consultations and digital tools to improve access to health services has been recognized before [8–10]. However, technical, administrative, financial, and professional barriers have limited the uptake and use of remote consultations [11, 12]. The COVID-19 pandemic has prompted the use of innovative ways to continue outpatient care for patients with NCDs, resulting in a sharp increase in the use of remote counselling [13]. Telemedicine has been acknowledged as one of the solutions to ensure health monitoring and disease management for patients with NCDs and to protect vulnerable populations with a high risk of infection and negative health outcomes [14].

Before the state of emergency, in the pre-crisis phase, remote counselling was not routinely provided and covered by public health funding in Latvia. The decisions to reimburse GPs and specialists for remote consultations were passed on the 18th and 27th of March 2020, respectively [15, 16]. The use of formal remote consultations for patient care was a new experience for clinicians in Latvia’s primary and secondary health care practice. This study was designed to evaluate the changes in the structure and volume of healthcare consultations for patients with selected NCDs by paying particular attention to the roles and conditions of remote health services.

This study aimed to describe the dynamics of consultations and the volume of remote consultations provided for patients with particular NCDs and to explore clinicians’ experiences of providing remote consultations during the first wave of the COVID-19 pandemic in Latvia.

## Methods

The study was carried out as part of the VPP-COVID-2020/1–0011 project of the National Research programme to assess the impact of COVID-19 on health care and public health in Latvia.

### Study design

This study utilized a mixed-method approach using a convergent design as described by Creswell and Clark [17]. The study analysed quantitative data from the National Health Services (NHS) and employed inductive thematic analysis of qualitative data from semi-structured interviews with clinicians. Both data sets were collected and analysed in parallel. Integration through narrative was used at the interpretation and reporting level; the findings were analysed separately, using a contiguous approach, and merged in the discussion [17, 18].

### Quantitative study

Data from the Latvia NHS were used to quantitatively illustrate the change in health service provision. All residents of Latvia are entitled to state-funded health care, and the NHS is responsible for contracts with health care providers and payments for publicly funded services. The use of private health care is not recorded in the NHS data and, therefore, this variable was not included in the analysis. When any service is reported to the NHS, the main diagnosis is indicated according to the 10th revision of the International Statistical Classification of Diseases and Related Health Problems (ICD-10). The recording of comorbidities is optional; there is no identifier used for suspected or confirmed cases of the disease. To characterize the care of NCDs, the following conditions (ICD-10 codes) were selected: 1) breast cancer (C50) in women and prostate cancer (C61) in men; 2) type 1 and type 2 diabetes (E10 and E11); 3) hypertension, coronary heart disease and congestive heart failure (I10, I20-I25, and I50); and 4) chronic obstructive pulmonary disease (COPD) and asthma (J44 and J45). All records of patient consultations in the entire adult population with these codes were extracted from the NHS Database of Outpatient Services alongside with the speciality of the medical professional who provided the service. Records from GPs were analysed for all abovementioned conditions, whereas specialists were selected for each group of diseases as follows: surgeons for breast cancer, urologists for prostate cancer, endocrinologists for diabetes, cardiologists for hypertension, coronary heart disease, and heart failure, and pulmonologists and/or allergists for chronic obstructive pulmonary disease and asthma.

The main focus was on the number of consultations provided in the first half of 2020; nevertheless, the same period in 2019 was also included to assess the expected pattern of service use. The first weeks were excluded because of their different lengths, and the New Year holidays as well as the last weeks in June were excluded because they were public holidays. Thus, three main periods were defined: Period I, before the emergency (the weeks of January 6 to March 15 in 2020 were compared with the weeks of January 7 to March 17 in 2019); Period II, when restrictions were implemented (the weeks of March 16 to May 10 in 2020 were compared with the weeks of March 18 to May 12 in 2019); and Period III, when restrictions, including consultations with specialists, were gradually lifted (the weeks of May 11 to June 21 in 2020 were compared with May 13 to June 23 in 2019). Period II also covered the Eastern Holidays, as well as public holidays on the 1st and 4th of May in both years. The adult population of Latvia on the 1st of January in 2019 and 2020 was used to calculate the rate of service use. Because of the different lengths of the studied periods, the consultation rate was calculated as the number of consultations in the given period per 100,000 persons per day (population size multiplied by the number of days in the given period). The proportion of remote consultations was calculated as the number of remote consultations in the period divided by the total number of consultations in that period. OpenEpi software [19] was used to estimate 95% confidence intervals (CIs) for rates (Mid-P exact test), rate differences (Byar’s method) and proportions (Wilson Score Interval).

### Qualitative study

#### Participants

Purposive maximum variation sampling was employed across clinicians to ensure the spread and heterogeneity of the clinicians’ views.

A purposive maximum variation sampling ensures heterogeneity of research participants with a wide range of variation across different key criteria (age, gender, experience, region, discipline areas etc.) [20] to ensure the maximum variability within the data relevant to the phenomenon under examination [21].

Qualitative studies aim to identify the qualitatively different patterns observed in data rather than to quantify those patterns [22]. The question of sample size in a qualitative study can be difficult, as there are no precise guidelines in this area [23] and a broad range of suggestions of the numbers of participants needed for qualitative interviews are provided [22]. Generally, a sample size of between 15 and 30 individual interviews is mentioned as adequate in qualitative research striving to identify patterns across primary data-set [24]. The concept of ‘saturation’ is an appropriate tool for determining sample size in qualitative research [24].

Participants were selected according to the following sampling criteria: age; gender; place of practice (city or suburb/rural area); level of health care (primary or secondary); clinician type (GP or specialist physician); type of health care facility (ambulatory (outpatient care centre), hospital or both); and clinician work experience (in years). Table 1 presents the demographic data and characteristics of the study participants.

**Table 1:** Characteristics of the interviewed participants (*n* = 34)

Characteristics	n	%
**Level of health care and speciality**		
Primary care: general practitioner	19	55.88
Secondary care: specialist physician	15	44.12
cardiologist	3	20.00
oncologist	3	20.00
internist	3	20.00
endocrinologist	3	20.00
pulmonologist	3	20.00
**Type of health care facility (institution)**		
only ambulatory	22	64.70
only hospital	6	17.65
both ambulatory and hospital	6	17.65
**Place of practice**		
republican cities	22	64.70
other	12	35.30
**Clinician work experience (in years)**		
< 10	4	11.76
10–20	11	32.35
21–40	13	38.24
40<	6	17.65
**Age**		
25–45	12	35.30
46–65	16	47.05
66<	6	17.65
**Gender**		
women	28	82.35
men	6	17.65

Participants were recruited via professional associations for healthcare workers and health care institutions. Recruitment began with the development of an official informative invitation letter for clinicians to participate in the study. The letter was then distributed to the relevant professional associations and health care institutions with a request to inform clinicians about the study. A total of thirty-four clinicians were interested in participating in the study and agreed to the interview.

#### Data collection

The semi-structured interview method was used for qualitative data collection in the present research. Before the interviews, an interview guide was developed and piloted. The interview guide included questions on the clinicians’ experiences of adapting practice management to the COVID-19 context; experiences of providing care for NCDs patients in an emergency due to COVID-19; perceptions of patients’ health needs; attitudes and beliefs about remote consulting; and perceptions of the benefits and risk factors associated with the care of NCD patients in a state of emergency, including the risks and benefits of remote consultation.

Data were collected between August 2020 and November 2020.

Six researchers who had no prior relationship with the research participants participated in the interview process.

The interviews were conducted individually; i.e., one researcher and one research participant met either in person or remotely via video communication or phone depending on the interviewee’s preference and the epidemiological situation in the country. Of the 34 interviews conducted, 20 were face-to-face interviews, and 14 were remote interviews (via phone, Skype, or Zoom). All face-to-face interviews were undertaken at the different health care centres where the research participants worked. No one except the participant being interviewed and the interviewer was present at the interviews. One semi-structured interview was conducted with each study participant. The researchers did not observe the need for repeated interviews.

Before each interview, written consent was provided, and the researcher repeated the purpose of the study. Participation in the study was completely voluntary. The participants were informed that they could withdraw from the interview at any time for any reason.

The interviews lasted between 35 and 90 min and were audio-recorded with participant permission.

#### Data analysis

The interviews were transcribed verbatim in preparation for the analysis.

To explore a broad scope of participant experiences, inductive thematic analysis was performed with the transcriptions, and the data were analysed manually using a six-step method as described by Braun and Clarke [25]: 1) familiarisation with data, 2) generating initial codes, 3) searching for emerging themes, 4) reviewing the themes, 5) defining and naming the themes, and 6) writing up the analysis and producing the report. Each interview transcript was coded independently by two researchers of the research team (MK and IS1). The team members discussed emergent themes, agreed on a final version of the list of general themes and subsequent sub-themes, and interpreted them.

Inductive thematic analysis was selected because this method encourages the development of themes exclusively based on the data as opposed to the adjustment of themes to a pre-selected thematic framework or predetermined theory [25, 26].

## Results

### Quantitative findings

**Fig. 1:**
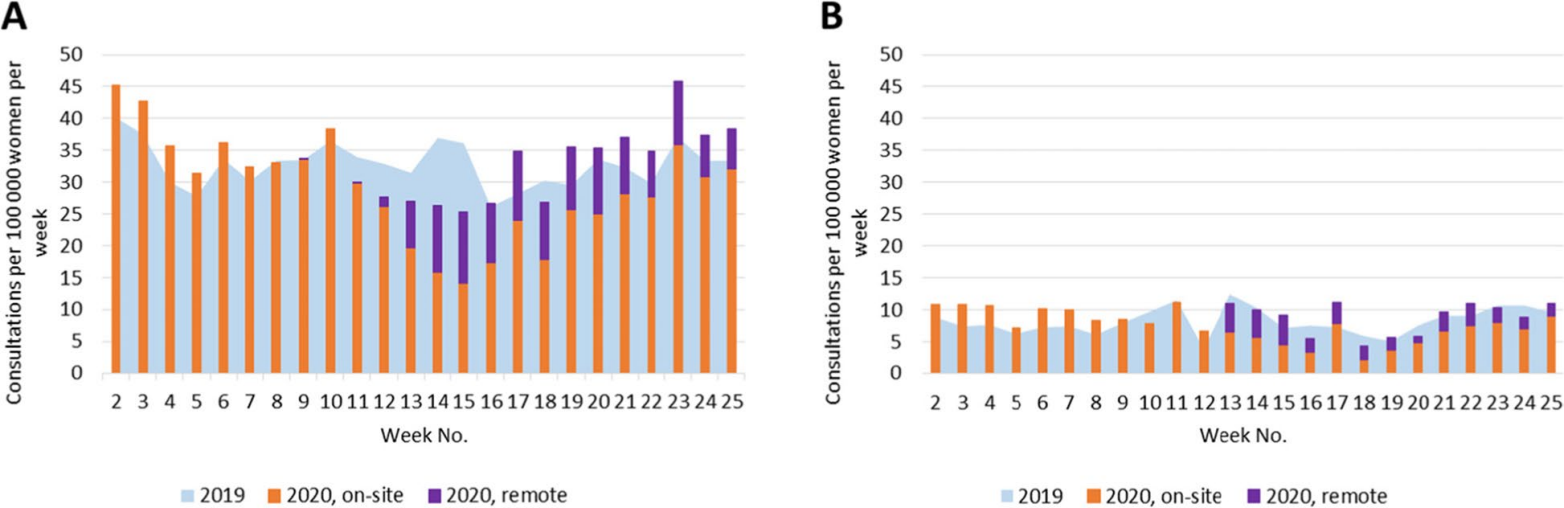
Number of consultations (disease code C50) per women population provided by: **A** - GPs; **B** – surgeons

**Fig. 2:**
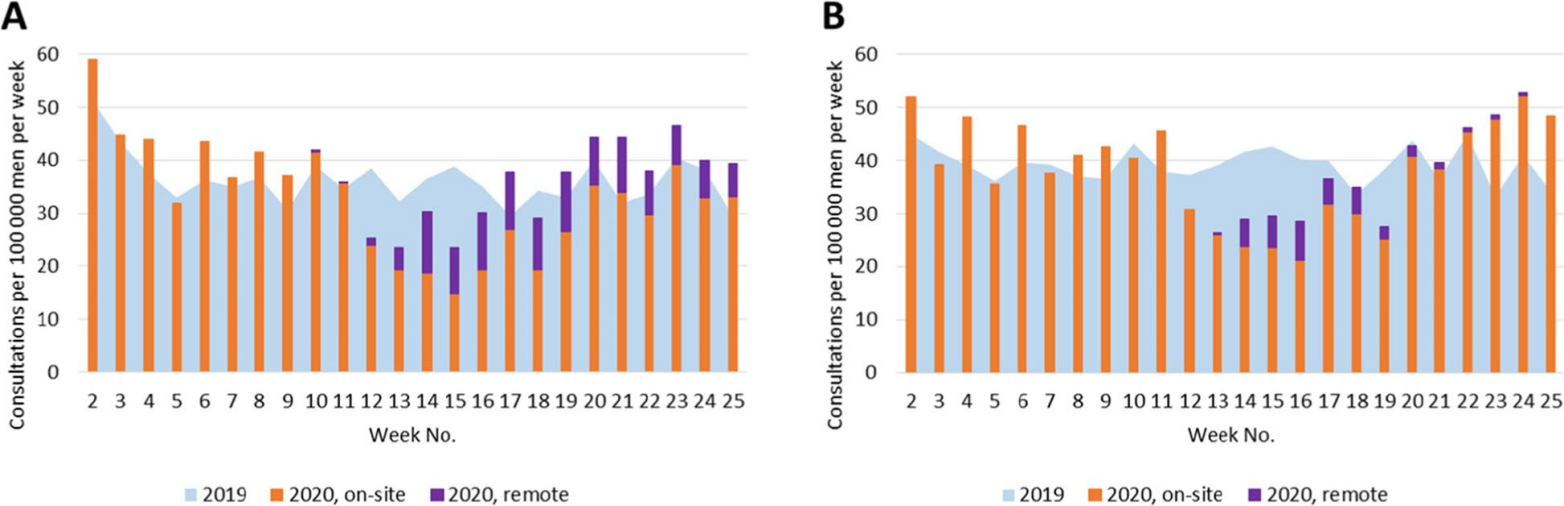
Number of consultations (disease code C61) per men population provided by: **A** - GPs; **B** – urologists

**Fig. 3:**
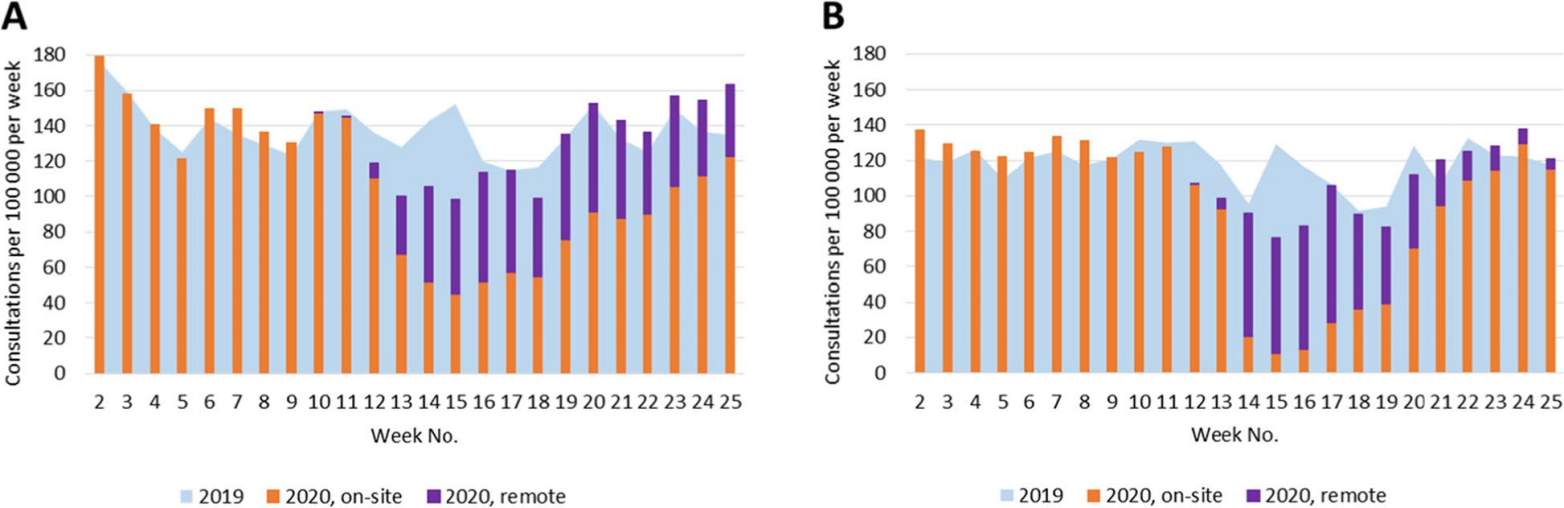
Number of consultations (disease codes E10 and E11) provided by: **A** - GPs; **B** – endocrinologists

**Fig. 4:**
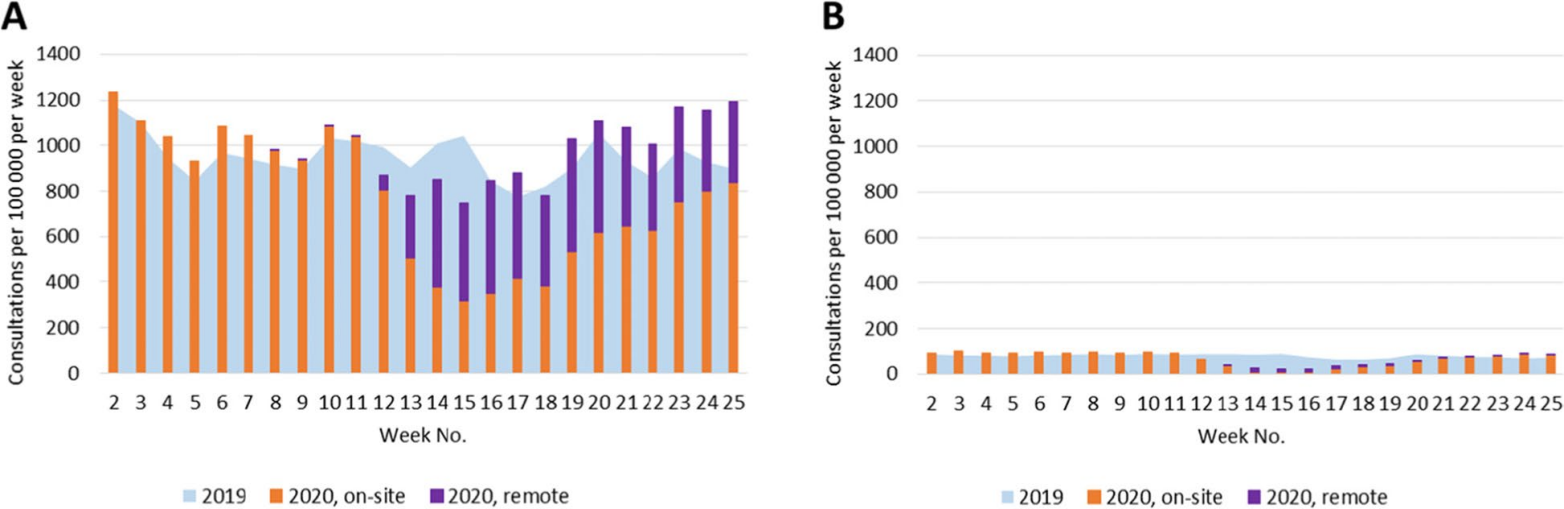
Number of consultations (disease codes I10, I20-I25, and I50) provided by: **A** - GPs; **B** – cardiologists

**Fig. 5:**
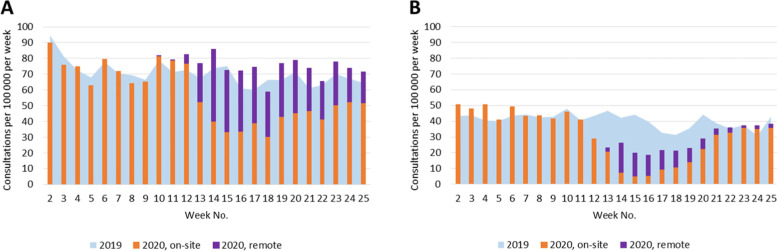
Number of consultations (disease codes J44 and J45) provided by: **A** - GPs; **B** – pulmonologists/allergists

During the first wave of the COVID-19 pandemic in 2020, the state of emergency and restriction of health care services affected the number of consultations provided for patients with selected chronic conditions by both GPs and specialists (Figs. 1, 2, 3, 4, 5). Among the GPs (Table 2), the highest relative decrease in the number of consultations occurred for prostate cancer and diabetes. When compared with Period I of 2020, Period II of 2020 had approximately 29 and 24% fewer consultations for prostate cancer and diabetes, respectively. In absolute numbers, consultations for prostate cancer decreased by 1.71 consultations per 100,000 persons per day, and consultations for diabetes decreased by 4.99 consultations per 100,000 persons per day. These two conditions also had the most significant relative decreases in consultations compared to those in the same period in 2019 – approximately 15%. The exceptions were COPD and asthma – the rate of consultations for these NCDs did not change between the three periods in 2020 and, in Periods II and III of 2020, the rate of consultations was even higher than those in the corresponding periods in 2019.

**Table 2:** Total number of consultations provided by general practitioners per population

	No. of consultations per 100,000 population^a^ per day (95% CI)			
	2019	2020	Difference between 2020 and 2019, *p*-value	Difference within 2020, p-value
*C50*				
Period I-III	4.69 (4.57; 4.80)	4.87 (4.76; 4.99)	0.18 (0.02; 0.35), 0.025	
Period I	4.81 (4.63; 4.99)	5.12 (4.94; 5.31)	0.32 (0.06; 0.57), 0.014	
Period II	4.49 (4.30; 4.69)	4.12 (3.93; 4.30)	−0.38 (− 0.64; − 0.11), 0.005	
Period III	4.75 (4.52; 4.98)	5.46 (5.22; 5.71)	0.71 (0.38; 1.04), < 0.001	
Period II vs Period I				−1.01 (−1.27; −0.75), < 0.001
Period III vs Period II				1.34 (1.04; 1.65), < 0.001
Period III vs Period I				0.33 (0.03; 0.64), 0.031
*C61*				
Period I-III	5.18 (5.05; 5.31)	5.39 (5.26; 5.53)	0.22 (0.03; 0.41), 0.022	
Period I	5.39 (5.19; 5.61)	5.95 (5.73; 6.17)	0.55 (0.25; 0.85), < 0.001	
Period II	4.97 (4.75; 5.19)	4.24 (4.03; 4.45)	−0.73 (− 1.03; − 0.43), < 0.001	
Period III	5.09 (4.83; 5.36)	6.02 (5.74; 6.31)	0.93 (0.54; 1.31), < 0.001	
Period II vs Period I				−1.71 (−2.01; − 1.41), < 0.001
Period III vs Period II				1.78 (1.43; 2.13), < 0.001
Period III vs Period I				0.07 (−0.29; 0.43), 0.703
*E10, E11*				
Period I-III	19.67 (19.49; 19.84)	19.35 (19.18; 19.52)	−0.32 (− 0.56; − 0.08), 0.010	
Period I	20.42 (20.15; 20.69)	20.82 (20.55; 21.09)	0.40 (0.02; 0.79), 0.040	
Period II	18.64 (18.35; 18.93)	15.82 (15.56; 16.09)	−2.82 (−3.21; − 2.42), < 0.001	
Period III	19.78 (19.44; 20.13)	21.59 (21.23; 21.95)	1.81 (1.31; 2.31), < 0.001	
Period II vs Period I				−4.99 (−5.37; − 4.61), < 0.001
Period III vs Period II				5.77 (5.32; 6.21), < 0.001
Period III vs Period I				0.77 (0.32; 1.22), < 0.001
*I10, I20-I25, I50*				
Period I-III	135.64 (135.20; 136.10)	142.79 (142.30; 143.30)	7.16 (6.51; 7.80), < 0.001	
Period I	140.63 (139.90; 141.30)	149.77 (149.00; 150.50)	9.15 (8.13; 10.16), < 0.001	
Period II	130.17 (129.40; 130.90)	121.27 (120.50; 122.00)	−8.90 (−9.96; −7.84), < 0.001	
Period III	134.62 (133.70; 135.50)	159.86 (158.90; 160.80)	25.24 (23.92; 26.57), < 0.001	
Period II vs Period I				− 28.50 (−29.54; −27.46), < 0.001
Period III vs Period II				38.59 (37.36; 39.81), < 0.001
Period III vs Period I				10.09 (8.87; 11.31), < 0.001
*J44, J45*				
Period I-III	10.06 (9.94; 10.18)	10.62 (10.50; 10.75)	0.56 (0.39; 0.74), < 0.001	
Period I	10.71 (10.52; 10.91)	10.63 (10.44; 10.83)	−0.08 (− 0.36; 0.19), 0.557	
Period II	9.70 (9.49; 9.91)	10.70 (10.48; 10.92)	1.00 (0.70; 1.31), < 0.001	
Period III	9.46 (9.22; 9.70)	10.50 (10.25; 10.76)	1.04 (0.70; 1.39), < 0.001	
Period II vs Period I				0.07 (−0.22; 0.36), 0.648
Period III vs Period II				−0.20 (− 0.53; 0.14), 0.246
Period III vs Period I				−0.13 (− 0.45; 0.19), 0.428

^a^ Female and male populations for breast and prostate cancer consultations, respectively

Regarding specialist care (Table 3), the highest decrease in Period II of 2020 compared with that in Period I was observed for consultations provided by cardiologists for conditions such as hypertension, coronary heart disease, and heart failure. In Period II of 2020, the cardiologists provided 8.29 fewer consultations per 100,000 persons per day than in Period I of 2020 (61% relative decrease) and 5.86 fewer consultations per 100,000 persons per day than in Period II of 2019 (53% relative decrease). Unlike the consultations provided by GPs, for the consultations provided by specialists, patients with COPD and asthma experienced the second-largest decrease in the consultation rate: in Period II of 2020, pulmonologists (and/or allergists) provided 50% fewer consultations than in Period I of 2020 and 43% fewer consultations than in Period II of 2019. However, the rate of consultations for patients with diabetes provided by endocrinologists decreased less: in Period II of 2020, it was 28% lower than in Period I of 2020 and 17% lower than in Period II of 2019.

**Table 3:** Total number of consultations provided by general practitioners per population

	No. of consultations per 100,000 population^a^ per day (95% CI)			
	2019	2020	Difference between 2020 and 2019, *p*-value	Difference within 2020, *p*-value
*C50: surgeons*				
Period I-III	1.17 (1.11; 1.23)	1.28 (1.22; 1.34)	0.11 (0.03; 0.20), 0.006	
Period I	1.14 (1.06; 1.23)	1.36 (1.27; 1.46)	0.22 (0.09; 0.35), < 0.001	
Period II	1.06 (0.97; 1.16)	1.13 (1.10; 1.23)	0.07 (−0.06; 0.20), 0.306	
Period III	1.35 (1.23; 1.47)	1.35 (1.23; 1.48)	0.00 (−0.17; 0.17), 0.983	
Period II vs Period I				−0.23 (− 0.36; − 0.10), < 0.001
Period III vs Period II				0.22 (0.06; 0.37), 0.005
Period III vs Period I				−0.01 (− 0.17; 0.14), 0.874
*C61: urologists*				
Period I-III	5.61 (5.48; 5.75)	5.66 (5.52; 5.80)	0.05 (−0.14; 0.24), 0.609	
Period I	5.65 (5.44; 5.87)	6.13 (5.91; 6.36)	0.48 (0.17; 0.78), 0.002	
Period II	5.59 (5.36; 5.83)	4.35 (4.14; 4.56)	−1.24 (− 1.56; − 0.93), < 0.001	
Period III	5.57 (5.30; 5.84)	6.63 (6.34; 6.93)	1.07 (0.66; 1.47), < 0.001	
Period II vs Period I				− 1.78 (−2.09; − 1.48), < 0.001
Period III vs Period II				2.28 (1.92; 2.65), < 0.001
Period III vs Period I				0.50 (0.13; 0.87), 0.008
*E10, E11: endocrinologists*				
Period I-III	16.88 (16.72; 17.04)	16.39 (16.24; 16.55)	−0.48 (− 0.71; − 0.26), < 0.001	
Period I	17.47 (17.23; 17.72)	18.23 (17.97; 18.49)	0.76 (0.40; 1.11), < 0.001	
Period II	15.75 (15.48; 16.01)	13.10 (12.86; 13.34)	−2.65 (− 3.01; − 2.29), < 0.001	
Period III	17.39 (17.07; 17.71)	17.73 (17.41; 18.06)	0.34 (−0.12; 0.80), 0.144	
Period II vs Period I				−5.13 (− 5.48; − 4.78), < 0.001
Period III vs Period II				4.63 (4.23; 5.04), < 0.001
Period III vs Period I				−0.50 (− 0.91; − 0.08), 0.019
*I10, I20-I25, I50: cardiologists*				
Period I-III	11.49 (11.36; 11.62)	10.20 (10.07; 10.32)	−1.29 (− 1.47; − 1.11), < 0.001	
Period I	12.09 (11.88; 12.30)	13.55 (13.33; 13.78)	1.46 (1.16; 1.76), < 0.001	
Period II	11.13 (10.90; 11.35)	5.27 (5.11; 5.42)	−5.86 (− 6.13; − 5.59), < 0.001	
Period III	10.95 (10.70; 11.21)	11.17 (10.92; 11.44)	0.22 (− 0.14; 0.58), 0.236	
Period II vs Period I				−8.29 (− 8.56; − 8.02), < 0.001
Period III vs Period II				5.91 (5.61; 6.21), < 0.001
Period III vs Period I				−2.38 (− 2.72; − 2.04), < 0.001
*J44, J45: pulmonologists and/or allergist*				
Period I-III	5.80 (5.71; 5.89)	5.05 (4.97; 5.14)	−0.75 (− 0.87; − 0.62), < 0.001	
Period I	6.13 (5.98; 6.28)	6.49 (6.34; 6.64)	0.36 (0.15; 0.57), < 0.001	
Period II	5.63 (5.48; 5.80)	3.25 (3.12; 3.37)	−2.39 (− 2.59; − 2.19), < 0.001	
Period III	5.46 (5.28; 5.64)	5.07 (4.89; 5.24)	−0.40 (− 0.65; − 0,14), 0.002	
Period II vs Period I				−3.24 (− 3.44; − 3.05), < 0.001
Period III vs Period II				1.82 (1.61; 2.03), < 0.001
Period III vs Period I				−1.42 (− 1.65; − 1.19), < 0.001

^a^ Female and male populations for breast and prostate cancer consultations, respectively

Under the restrictions in 2020, a large proportion of consultations were provided remotely (Figs. 1, 2, 3, 4, 5, Table 4). In Period II of 2020, when the strongest restrictions were in place, the GPs provided approximately one-third of cancer-related consultations and almost half of consultations for the other conditions remotely. Among the specialists, endocrinologists had the highest proportion of remote consultations (up to 72.0% in Period II of 2020), while urologists had the lowest (16.4%). Nevertheless, as soon as face-to-face consultations with specialists were allowed (in Period III of 2020), remote specialist consultations became rare, while family doctors, albeit to a lower extent, continued using this approach.

**Table 4:** Proportion of remote consultations in 2020 by diagnosis and specialist

	% of remote consultations out of total No. (95%CI)	
*General practitioners*		
	March 23 – May 10	May 11 – June 21
C50	33.7 (31.5; 35.9)	21.6 (19.8; 23.5)
C61	31.6 (29.3; 34.0)	19.2 (17.4; 21.1)
E10, E11	47.5 (46.6; 48.4)	32.9 (32.1; 33.7)
I10, I20-I25, I50	51.1 (50.8; 51.5)	36.2 (35.9; 36.5)
J44, J45	47.5 (46.4; 48.6)	34.8 (33.7; 36.0)
*Specialists*		
	March 30 – May 10	May 11 – June 21
C50: surgeons	40.9 (36.1; 45.9)	24.1 (20.4; 28.1)
C61: urologists	16.4 (14.5; 18.6)	1.9 (1.4; 2.7)
E10, E11: endocrinologists	72.0 (71.0; 72.9)	15.1 (14.4; 15.7)
I10, I20-I25, I50: cardiologists	35.9 (34.2; 37.7)	4.1 (3.6; 4.5)
J44, J45: pulmonologists and/or allergists	59.6 (57.4; 61.8)	9.1 (8.1; 10.1)

### Qualitative findings

Five major themes and thirteen sub-themes emerged through inductive thematic analysis, and they are presented in Table 5.

**Table 5:** Themes and sub-themes identified in the thematic analysis

Theme	Sub-theme
1. Adjusting in a time of confusion and fear	Adapting through swift changes in regulations
	Lack of disease-specific information
	The burden of paperwork
	Fear as a barrier for consultation
2. Remote consultations: safety versus availability	Improved efficiency and availability
	The perpetual dilemma of the consultation format
	Fear of missing relevant information
3. Sacrifice and loss of privacy	Patient data protection and privacy issues
	Clinicians’ separation of their private lives from work
4. Advantages and disadvantages of communication technologies	Technological support
	eHealth system
5. Different form of communication and a health literacy challenge	Increased responsibility
	Health literacy gaps as a risk factor for remote consultations

#### Theme 1. Adjusting in a time of confusion and fear

This theme describes the sense of confusion, sense of uncertainty, and fear of rapid change toward the unknown caused by the COVID-19 pandemic, as experienced by clinicians. The situation seemed insecure and demanding for several reasons, as described below.

##### Adapting through swift changes in regulations

The necessity to adapt to overnight changes to health care provision due to the declaration of an emergency was commonly mentioned by a variety of clinicians from different specialities but was emphasized most by the GPs.

*“Actually, I didn’t even have much time to think about it. What is going on, I found out on Thursday evening, March 12*^*th*^*, after appointment time. Basically, around seven o’clock in the evening, calls from patients started to come in [...] And it was like … what’s going on? What to do? What will happen?”(Participant 14, GP, woman, age 46-65)*

Both GPs and specialists reported high levels of stress caused by the continuous day-to-day changes in regulations and algorithms regarding work organization and treatment protocols. Concerns about possibly missing relevant new information were also experienced.

##### Lack of disease-specific information

The lack of unified information on algorithms for consultations and treatment approaches for patients with particular diseases in the context of COVID-19 was dominantly reported by the specialists. The doctors felt rising anxiety due to the increased responsibility associated with treatment decisions regarding patients with chronic diseases and COVID-19 infection.

*“Those specific things for each ward, for each patient group, those we needed to come up with on-site.” (Participant 32, internist, woman, age 25-45)*

##### The burden of paperwork

Remote consultations becoming an official format for the doctor-patient encounter came with new registration requirements. As clinicians reported, remote consultations already existed but as an unspecified practice. The increased amount of time needed to perform and then register the remote consultations in comparison with on-site consultations was mentioned as one of the new adaptation challenges.

##### Fear as a barrier for consultation

Patients’ fear of becoming infected with COVID-19 was commonly mentioned as a reason why patients did not seek medical help promptly. The doctors observed that patients downplayed changes in their health statuses, which led to delayed diagnosis and treatment.

*“I had one case when a chronic patient on the phone said that everything was the same. That was after heart surgery, with chronic heart failure, but when we could meet for on-site consultation, it turned out that she had a lung tumour with metastasis. If we would have met during the period of restrictions, it could have been discovered earlier. She called the ambulance only when it got really bad.” (Participant 13, GP, woman, age 46-65)*

#### Theme 2. Remote consultations: safety versus availability

In the context of the limited availability of healthcare services, remote consultation became an adequate way to ensure patient access to a clinician. However, in addition to promising opportunities, remote consultations also led to some confusion and concerns regarding the quality of provided services and created a new dilemma for clinicians.

##### Improved efficiency and availability

Improved efficiency in planning family physicians’ office work and increased availability to those in need of on-site consultations were observed. Family physicians reported a higher degree of freedom in the decision-making process regarding the consultation format, as well as a decrease in unnecessary on-site consultations.

*“People went to the doctor only when they needed to.” (Participant 6, GP, woman, age 25-45)*

##### The perpetual dilemma of the consultation format

Determining the necessity for on-site consultation via remote consultation was highlighted as one of the greatest challenges. Doctors with no prior preparation or guidelines were required to remotely determine patients’ need for an on-site consultation. The GPs reported higher confidence in making decisions about their “regular” patients whose health needs and medical conditions were better known.

##### Fear of missing relevant information

The clinicians’ mentioned a fear of missing relevant information during remote consultations. They repeatedly expressed their anxiety regarding their limited ability to adequately evaluate a patient’s health condition without performing a physical examination.

*“There is no way of auscultating [the lungs] on phone” (Participant 31, pulmonologist, woman, age 46-65)*

Additionally, practical knowledge, skills, and guidelines on how to gather the necessary information through remote consultation were lacking, which increased physicians’ concerns.

#### Theme 3. Sacrifice and loss of privacy

The rapid start of the use of remote consultation as a routine healthcare component created personal data security and privacy issues for both doctors and patients.

##### Patient data protection and privacy issues

During the period of extensive remote consultations, without a previously established framework for providing remote healthcare services, patient data protection and privacy issues were highlighted as problems by the clinicians. The doctors indicated that they were forced to use various information channels, such as WhatsApp, Facebook, and email, to exchange sensitive information about patients’ health. The use of such channels in turn raised the issue of patient data security.

##### Clinicians’ separation of their private lives from work

The GPs reported a fading line between their professional and private lives due to patient habituation to searching for doctors’ advice at any time. The GPs shared their experiences of receiving and answering patient phone calls after working hours and on weekends.

*“At night [patients] could call; there were even patients who called because they got runny noses.” (Participant 7, GP, woman, age 46-65)*

#### Theme 4. Advantages and disadvantages of communication technologies

With the initiation of remote consultations, technological challenges emerged. The state of emergency intensified already existing problems in the eHealth system and identified new needs for communication technologies. In addition, the clinicians noted promising possibilities for the future development of consultation formats as well as the value of available e-resources.

##### Technological support

Overall, a positive attitude towards the development of remote consultation in the future was expressed, with an emphasis on problematic issues that required improvement to successfully provide remote consultations.

The doctors acknowledged that the information and communication technologies available in their practice were not always well suited to remote patient consultation. The most frequently mentioned technical problems were related to a lack of qualitative tools. According to physicians’ observations, patients also faced some technical limitations that affected the quality of consultation, e.g., the ability to perform a remote visual assessment of a patient.

*“To be able to see the patients, that is a thing to work on.” (Participant 24, pulmonologist, woman, age 25-45)*

##### eHealth system

The functionality of the eHealth system was a widely discussed subject. Regarding the eHealth system, mostly criticisms were expressed, with an emphasis on poor experiences with the slow-working system lacking valuable functions.

*“The eHealth in Latvia doesn’t work. It works only for medication e-prescriptions and for electronic sick leave request forms.” (Participant 2, GP, man, age 66<)*

*“It [working in eHealth] is like requiring iPhone functions from a pocket calculator.” (Participant 34, cardiologist, man, age 46-65)*

However, the clinicians mentioned that the existing functions, although few, were helpful for work organization and proved to be valuable.

*“.. now the system justified itself.” (Participant 14, GP, woman, age 46-65)*

Many of the clinicians talked about the need for enhancements to the eHealth system that would be particularly important during the pandemic. The most common suggestion was the creation of a fully functioning eHealth system where all patients’ medical records could be gathered and readily available for clinicians during consultations.

#### Theme 5. Different form of communication and a health literacy challenge

The change in the form of doctor-patient communication increased the patient’s responsibility and participation in his/her health care. With the promising evolution of the doctor-patient relationship, the newly required skills needed for successful implementation of remote consultations highlighted the oftentimes insufficient level of health literacy.

##### Increased responsibility

Clinicians reported an increase in patients’ active participation in their health monitoring and protection. For example, patients measured their blood sugar levels and blood pressure and, in physicians’ opinion, were more responsible and disciplined.

##### Health literacy gaps as a risk factor for remote consultations

The necessity for higher levels of patient involvement revealed the insufficient health literacy of some patients, which can pose a risk to patients’ health. The clinicians reported patients’ misuse of prescribed medications and their inability to describe their symptoms to the doctor during remote consultation as some of the manifestations of insufficient patient health literacy in the context of remote consultation.

## Discussion

The COVID-19 pandemic abruptly changed the context in which the health care system operated and posed new challenges for the continuity of health care provision for patients with NCDs [27, 28]. The emergency during the first wave of the COVID-19 pandemic in Latvia affected not only the number and structure of consultations for patients with selected NCDs but also new health care counselling practices among health care providers. A significant decrease in health care consultations by both GPs and specialists for selected NCDs occurred when the restrictions on planned health care services were most severe (the weeks of March 16 to May 10, 2020). The provision of consultations for cancer-related conditions was less affected; however, counselling for breast and prostate cancer by both GPs and specialists declined, even though cancer care was exempted from the health service restrictions. The qualitative findings indicate that patients’ fear of contracting COVID-19 was one of the factors contributing to their decision to delay consulting with a doctor even as their symptoms worsened or new symptoms manifested.

Remote consultations, which accounted for a significant share of the total number of consultations during the first wave of the COVID-19 pandemic, were essentially a new type of health care service in Latvia. There are countries where telemedicine services have been around for decades, such as the United Kingdom where primary care providers have been encouraged to develop new, flexible models of patient access, including remote types of consultation in the 2022 GP Action Plan [29]. In Latvia, reporting on remote consultations to the NHS was subserved by reimbursement, which was introduced only in the second half of March 2020. In spring 2020, 31.6–51.1% of GPs consultations for particular NCDs in Latvia were provided remotely. Other studies reported various results with remote consultations making 34–89% of all consultations provided by GPs, with a higher proportion reached in health care systems with pre-established and routinely-used telemedicine [30, 31]. Some degree of underreporting of remote consultations in Latvia is very likely, particularly among GPs, because of the work intensity. The qualitative findings highlight increased time resources required to register remote consultations and eHealth functionality issues, which could hinder the reporting.

Despite the initiation of remote consultations, the total volume of consultations provided by both GPs and specialists was significantly lower for most of the selected NCDs compared with the same period in 2019, experiencing up to a 15% decrease in GP consultations for prostate cancer and diabetes and a 43% decrease in pulmonologist consultations. Moreover, when the restrictions on health services were lifted, the proportion of remote consultations declined, with a more significant decrease observed for specialist consultations (Table 4). Several emerging themes in qualitative findings illustrate obstacles for remote consultations to be seen as a routine form of patient consultation. One of the commonly mentioned complexities of remote consultations was the ability to make the right treatment decision without face-to-face contact and physical examination of a patient. Similar concerns, especially regarding patients with a new set of symptoms, were expressed in other studies [32]. Fewer concerns were raised regarding well-known patients. The findings in this study are consistent with previously published data indicating some reluctance among GPs to implement remote consultations due to concerns about medico-legal aspects and technical support; however, if these issues can be addressed, clinicians are open to the development of telemedicine in clinically appropriate situations [33–35]. The necessity for a well-established framework for remote consultations with easy-to-use administrative systems, clear guidelines and centralized technological support was noted by the clinicians in the interviews. These findings align with conclusions from other studies that indicate the necessity of a strategic approach and the involvement of all main stakeholders in the development of telemedicine services [36, 37]. Remote consultations must be considered a separate health care service, not an on-site consultation performed remotely, and must be planned and organized accordingly.

In particular, a necessity for enhancements in the eHealth system emerged as a separate theme in our findings with special significance regarding remote consultations. The development of an eHealth system in Latvia started in 2005, but the use of the central eHealth system is still voluntary for medical institutions. Since January 2018, electronic sick-leave certificates and prescriptions for state reimbursed pharmaceuticals have been mandatory. Health care institutions are obliged to provide online information on referrals for inpatient or hospital services, radiological examinations, outpatient examinations, and vaccinations [38]. Unfortunately, today, the eHealth system is affected by a broad variety of technical and technological problems, as well as legal and data security issues, resulting in the low commitment of healthcare organizers and providers to use the system. Detailed analysis of the problems faced by the eHealth platform has already been published elsewhere [39].

The COVID-19 pandemic has challenged health care systems globally [27, 40]. It has also shed light on the myriad of long-term health care problems in Latvia, e.g., the adversities in the development and operability of the eHealth system, the lack of clearly regulated telemedicine services and the low health and digital literacy of patients and healthcare providers, as revealed by our study findings described above. The need for the mentioned issues to be resolved has been more visible, as these issues play a critical role in ensuring timely access to appropriate healthcare for the Latvian population, not only in times of crisis but also beyond, especially in the remote parts of the country. The Latvian healthcare system and policymakers have recognized these realities and considered the lessons of this crisis by developing a basis for legislation on the provision, registration and compensation of remote consultations [41] and announcing the development of a new, modern, up-to-date eHealth platform [42]. Many issues remain to be addressed to fully solve the identified problems; nonetheless, by prompting the establishment of telemedicine services as a permanent and well-functioning part of the Latvian healthcare system in the future and by simultaneously demanding physical distancing and increased access to healthcare services, similar to other countries [43, 44], the COVID-19 crisis has unintentionally served as a powerful catalyst for fundamental long-awaited improvements in Latvian healthcare. However, in the future, the performance and effectiveness of implemented eHealth services, tools, and measures should be evaluated in detail.

### Limitations

This study has several limitations that should be taken into account. The analysis of data on provided health care consultations from the NHS database covers the whole population, but consultations that occurred in the private sector were not included. Data on the volume and dynamics of consultations provided in the private sector were not available and should be viewed as a potential confounder. Regarding services in the public sector and their reporting to the NHS, the validity of diagnostic codes is not systematically evaluated. In addition, these codes may be used for any tests or referrals related to suspected, not confirmed, conditions. Moreover, our analysis did not include consultations provided to patients with chronic diseases for reasons other than the selected disease codes. For example, NCD patients might have consulted GPs because of acute respiratory symptoms or any other health issues that were coded using other disease codes, and these consultations would therefore have not been analysed. Furthermore, GPs decision on the main diagnosis to be reported in routine consultations with multimorbid patients could be a confounder. Consequently, there is a possibility that consultation volume distribution in-between diagnoses analysed was affected since the recording of comorbidities is optional. However, the NHS database provides access to country-level data on health care services allowing the assessment of the impact of the COVID-19 emergency on the national health care system. Regarding the qualitative data, maximum variation sampling criteria did not include specialists and GPs working in a private sector without a contract with the NHS, as well as specialists representing other fields of medicine (dermatology, rheumatology, geriatrics etc.) therefore their perspective was not explored. All GPs and specialists who participated in the study were volunteers, and thus possibly more extroverted and willing to express their experiences, and more research-oriented. Qualitative interviews were performed a few months after the end of the first wave of the COVID-19 emergency. Therefore, the accuracy of the clinicians’ reports of their experiences might have been affected by recall bias. Nonetheless, the qualitative analysis enhanced our in-depth understanding of clinicians’ experiences of consultations in the context of the COVID-19 crisis and restrictions of health care services in Latvia.

## Conclusions

Disruptions of health care services for patients with NCDs decreased the total number of consultations provided by both GPs and specialists during the first wave of the COVID-19 pandemic in Latvia. The emergence of remote consultations as a separate health care service and its significant contribution to the total number of consultations provided during the period when restrictions were implemented reflect the need for this type of healthcare service for patients with NCDs. For remote consultations to be successful, telemedicine needs to be further developed and acknowledged by both, clinicians and patients, as a health service, improvements to the eHealth system need to be made, and a special framework for the provision of remote consultation needs to be created. Establishing the use of telemedicine can ensure the continuity of health care in public health crises and the availability of healthcare services, as well as promote the modernization of health care systems.

## Data Availability

The data that support the quantitative findings of this study are available from the NHS of Latvia but restrictions apply to the availability of these data, which were used under license for the current study, and so are not publicly available. Data are however available from the authors upon reasonable request and with permission of the NHS of Latvia. The datasets generated and/or analysed during the qualitative interviews of the current study are not publicly available due to the risk of disclosure of confidential information but are available from the corresponding author on reasonable request.

## Abbreviations

COPD: Chronic obstructive pulmonary disease
COVID-19: Coronavirus Disease 2019
EU: European Union
GP: General practitioner
NCD: Non-communicable disease
NHS: National Health Service
WHO: World Health Organisation
